# TNF superfamily member APRIL enhances midbrain dopaminergic axon growth and contributes to the nigrostriatal projection *in vivo*

**DOI:** 10.1016/j.expneurol.2017.09.007

**Published:** 2017-12

**Authors:** Thomas G. McWilliams, Laura Howard, Sean Wyatt, Alun M. Davies

**Affiliations:** Division of Molecular Biosciences, School of Biosciences, Cardiff University, Museum Avenue, Cardiff CF10 3AX, United Kingdom

**Keywords:** Axon growth, Midbrain dopaminergic neuron, Nigrostriatal projection, TNF superfamily, APRIL knockout mice

## Abstract

We have studied the role of the tumor necrosis factor superfamily member APRIL in the development of embryonic mouse midbrain dopaminergic neurons *in vitro* and *in vivo*. In culture, soluble APRIL enhanced axon growth during a window of development between E12 and E14 when nigrostriatal axons are growing to their targets in the striatum *in vivo*. *April* transcripts were detected in both the striatum and midbrain during this period and at later stages. The axon growth–enhancing effect of APRIL was similar to that of glial cell-derived neurotrophic factor (GDNF), but in contrast to GDNF, APRIL did not promote the survival of midbrain dopaminergic neurons. The effect of APRIL on axon growth was prevented by function-blocking antibodies to one of its receptors, BCMA (TNFRSF13A), but not by function-blocking antibodies to the other APRIL receptor, TACI (TNFRSF13B), suggesting that the effects of APRIL on axon growth are mediated by BCMA. *In vivo*, there was a significant reduction in the density of midbrain dopaminergic projections to the striatum in *April* −/− embryos compared with wild type littermates at E14. These findings demonstrate that APRIL is a physiologically relevant factor for the nigrostriatal projection. Given the importance of the degeneration of dopaminergic nigrostriatal connections in the pathogenesis and progression of Parkinson's disease, our findings contribute to our understanding of the factors that establish nigrostriatal integrity.

## 1 Introduction

Parkinson's disease is a neurodegenerative disease that affects about 1% of the population over the age of 60. It is characterized by progressive and disabling motor symptoms that are due to the degeneration and loss of the dopaminergic neurons of the substantia nigra pars compacta that project to the striatum (Lindholm et al., 2016). While defective trophic support appears to play no role in the pathogenesis or progression of the disease, several neurotrophic factors that sustain the survival of cultured midbrain dopaminergic neurons and enhance neurite outgrowth have been shown to be efficacious in animal models of Parkinson's disease. The most extensively studied neurotrophic factor for midbrain dopaminergic neurons is glia cell-derived neurotrophic factor (GDNF) (de Tassigny et al., 2015). GDNF was originally identified as a factor that promotes the survival of cultured midbrain dopaminergic neurons (Lin et al., 1993) and reduces the loss and degeneration of these neurons in multiple rodent and primate models of Parkinson's disease (Kordower and Bjorklund, 2013). However, mice lacking GDNF, which die shortly after birth because of renal agenesis, have an intact nigrostriatal system (Airaksinen and Saarma, 2002). Although a reduction in the number of midbrain dopaminergic neurons was initially reported in adult mice that possess a conditional null mutation of the *Gdnf* gene in the striatum (Pascual et al., 2008), a more recent and comprehensive study of multiple striatal *Gdnf* conditional deletion mouse lines has failed to find any abnormal phenotype affecting the midbrain dopaminergic neurons (Kopra et al., 2015). There have been multiple clinical trials of GDNF in Parkinson's disease with variable outcomes. While some open-label trials have reported modest symptomatic improvement, no efficacy has been reported in any extensive double-blind trial (Olanow et al., 2015). This large body of work on GDNF and limited studies of other factors that exert trophic actions on dopaminergic neurons highlight the need to identify additional factors that act on these neurons, especially those that exert physiological relevant trophic actions *in vivo*.

Here we examined the potential actions of APRIL (A Proliferation-Inducing Ligand, TNFSF13) on developing midbrain dopaminergic neurons. APRIL is a member of the TNF superfamily that was initially identified by its ability to promote tumor growth (Hahne et al., 1998), but is best characterized for its role in regulating lymphocyte survival and activation (Schneider, 2005). Recently, APRIL has been reported to enhance axon elongation, but not dendrite growth, from developing hippocampal pyramidal cells *in vitro* (Osorio et al., 2014). For this reason, we studied the potential effects of APRIL on the clinically important dopaminergic neurons of the midbrain. We show that recombinant APRIL enhances axon growth from cultured embryonic mouse midbrain dopaminergic neurons and that the projection of these neurons to the striatum is significantly impaired in APRIL-deficient embryos. Our findings reveal that APRIL is a physiologically relevant factor for the establishment of the dopaminergic nigrostriatal projection *in vivo*.

## 2 Materials and methods

### 2.1 Animals

This study was conducted on tissues obtained from CD1 mice (*Mus musculus*) and mice with a null mutation in either the *April* gene (Castigli et al., 2004) (gift from Raif Geha, Boston Children's Hospital, Harvard Medical School, Cambridge, MA, USA) or the *Bax* gene (Knudson et al., 1995) (gift from the late Stanley Korsmeyer), both backcrossed into a CD1 background. Breeding and housing was approved by the Cardiff University Ethical Review Board and was performed within the guidelines of the Home Office Animals (Scientific Procedures) Act, 1986.

### 2.2 Neuron culture

The ventral midbrains were dissected from litters of CD1 embryos from E10 to E14, using electrolytically sharpened tungsten needles in chilled L-15 medium (Gibco). To aid dissociation, isolated tissue pieces were digested with 0.05% trypsin (Worthington Biochemical Corp., New Jersey, USA) in Ca^2 +^/Mg^2 +^–free Hanks Balanced Salt Solution (Life Technologies, Paisley, UK) for 15 min at 37 °C. To inactivate the trypsin, the tissue was washed twice in Ham's F-12 medium (Life Technologies) containing 10% heat-inactivated horse serum (Life Technologies) and centrifuged at 2000 *rpm*. The tissue was resuspended in 1 ml culture medium comprised of 1:1 DMEM/Ham's F-12, 1 × serum-free N-2 supplement, 2% B27 minus AO (Life Technologies), 2 mM l-Glutamine (Life Technologies), 1 U/0.1 mg/ml Penicillin/Streptomycin (Sigma-Aldrich, Poole, UK). The tissue was gently triturated with P1000 and P200 pipettes to generate a dissociated single cell suspension. The cells were plated on a poly-ornithine/laminin substratum at a density of 50,000 cells per well in four-well dishes (Greiner Bio-One, Stonehouse, UK), and allowed to adhere for 30 min to 1 h at 37 °C, 5% CO_2_ prior to the addition of factors. The cultures were supplemented with the following factors as indicated: recombinant human GDNF (Merck-Millipore, Durham, UK), recombinant human *mega*APRIL, (initial experiments were performed with ALX-522-035-3010 from Alexis Biochemicals, Enzo Life Sciences, Exeter, UK and subsequently with multimeric recombinant human APRIL AG-40B-0017-3010 from Adipogen Life Sciences, Caltag MedSystems, Buckingham, UK). Function-blocking antibodies were rat monoclonal anti-BCMA and goat polyclonal anti-TACI (MAB5931 and AF1041 respectively, R&D Systems, Abingdon, UK). Cultures were pre-incubated with these function-blocking antibodies for 1 h prior to the addition of recombinant APRIL.

### 2.3 Immunocytochemistry

The culture medium was gently aspirated and the cultures were washed with PBS at 37 °C. Cells were fixed with either ice-cold methanol (MeOH) for 5 min or freshly-made 4% paraformaldehyde (Sigma-Aldrich) in 0.12 M Phosphate buffer, pH 7.2 for 12 min. The fixative was removed and after washing with PBS, the cultures were blocked for ~ 1 h at room temperature in 5% BSA containing 0.2% Triton X-100 (Sigma-Aldrich). The cultures were incubated overnight at 4 °C with primary antibody in PBS containing 1% BSA (Sigma-Aldrich) and were gently agitated on an orbital shaker. After extensive washing in PBS, the cultures were incubated with fluorophore-conjugated secondary antibody (Alexa Fluor or Dylight 488/594) in 1% BSA for 1 h in the dark at RT. Following serial washes in PBS, the nuclei were counterstained with DAPI (1:10,000 dilution; Life Technologies) for 3 min. The cultures were imaged with either a Zeiss Axiovert 200 Inverted Fluorescence microscope using Simple PCI software, or with a Zeiss LSM 510 Confocal laser Scanning Microscope. The following primary antibodies were used: rabbit polyclonal anti-tyrosine hydroxylase (1:650 - AB152, Millipore), sheep polyclonal anti-tyrosine hydroxylase (1:500 - AB1542, Millipore), mouse monoclonal anti-tyrosine hydroxylase (1:500 - MAB318, Millipore), rabbit polyclonal anti-APRIL (1:200 - ab64967, Abcam, Cambridge, UK), rabbit polyclonal anti-BCMA (1:200 - ab5972, Abcam, Cambridge, UK), rabbit polyclonal anti-β-III Tubulin (1:500 - ab18207 “neuron-specific clone Tu20”, Abcam, Cambridge, UK).

### 2.4 Analysis of axon length and neuronal survival

Immunocytochemistry was used to visualize TH-positive neurons or all neurons using anti-β-III tubulin antibodies. Axons were traced using NIH Image-J software and mean axon length was determined. Survival analysis of mDA neurons was conducted using a micro-island assay, as described (Planken et al., 2010). Statistical analyses of two or more conditions were conducted *via* one-way ANOVA followed by *post*-*hoc* analysis with Bonferroni correction. Pair-wise comparisons were made using Student's *t*-test.

### 2.5 Quantitative PCR

The levels of *April*, *Bcma* and *Th* mRNAs were quantified by RT-QPCR in dissected ventral midbrain, striatum, ventral midbrain and SCG, respectively, relative to a geometric mean of mRNAs for the house keeping enzymes glyceraldehyde phosphate dehydrogenase (GAPDH) and succinate dehydrogenase (SDHA). Total RNA was extracted from dissected tissues with the RNeasy Mini Lipid extraction kit (Qiagen, Crawely, UK), and 5 μl was reverse transcribed for 1 h at 45 °C using the AffinityScript kit (Agilent, Berkshire, UK) in a 25 μl reaction according to the manufacturer's instructions. 2 μl of cDNA was amplified in a 20 μl reaction volume using Brilliant III ultrafast QPCR master mix reagents (Agilent, Berkshire, UK). QPCR products were detected using dual-labelled (FAM/BHQ1) hybridization probes specific to each of the cDNAs (MWG/Eurofins, Ebersberg, Germany). The PCR primers were: *April* forward, 5′-CTG TCC TTC CTA GAT AAT G-3′ and reverse, 5′-CTA GTG ACA CTC TGA CAC-3′; *Bcma* forward, 5′-TGA CCA GTT CAG TGA AAG G-3′ and reverse, 5′-GGG TTC ATC TTC CTC AGC-3′; *Th* forward, 5′-CAG AGT TGG ATA AGT GTC A-3′ and reverse, 5′-CTC ACC CTG CTT GTA TTG -3′; *Gapdh* forward, 5′-GAG AAA CCT GCC AAG TAT G-3′ and reverse, 5′-GGA GTT GCT GTT GAA GTC-3′; *Sdha* forward, 5′-GGA ACA CTC CAA AAA CAG-3′ and reverse, 5′-CCA CAG CAT CAA ATT CAT-3′. Dual-labelled probes were: *April*, 5′-FAM-CAC CAA ATT CTC CTG AGG CT-BHQ1–3′; *Bcma*, 5′-FAM-CGT ACA CGG TGC TCT GGA TCT TCT T-BHQ1–3′; *Th*, 5′-FAM-CAC CAA GTT TGA CCC TGA CCT G-BHQ1–3′; *Gapdh*, 5′-FAM-AGA CAA CCT GGT CCT CAG TGT-BHQ1–3′; *Sdha*, 5′-FAM-CCT GCG GCT TTC ACT TCT CT-BHQ1–3′. Forward and reverse primers were used at a concentration of 150 nM each and dual-labelled probes were used at a concentration of 300 nM. PCR was performed using the Mx3000P platform (Agilent, Berkshire, UK) using the following conditions: 45 cycles of 95 °C for 12 s and 60 °C for 35 s. Standard curves were generated in every 96-well plate, for each cDNA for every real time PCR run, by using serial three-fold dilutions of either reverse transcribed adult mouse spleen RNA, or, in the case of *Th* cDNA, adult mouse brain total RNA (AMS Biotechnology, Abingdon, UK). Three to six separate dissections were performed for each age.

### 2.6 Analysis of the dopaminergic nigrostratal projection *in vivo*

The midbrain dopaminergic projection to the striatum was analysed using immunolabeling-enabled three-dimensional imaging of solvent-cleared organs (iDISCO) (Renier et al., 2014). Briefly, E14 *April* +/+ and *April* −/− embryos were fixed in 4% paraformaldehyde for 24 h and serially dehydrated in methanol/phosphate buffered saline (PBS). The samples were then bleached overnight in chilled 5% H_2_O_2_ to reduce tissue auto-fluorescence before being serially rehydrated in methanol/PBS containing 0.2% Triton X-100. The embryos were incubated in blocking solution (6% donkey serum, 20% DMSO, 0.2% Triton X-100, 0.3 M Glycine in PBS) for 72 h at 37 °C. After washing with PBS containing 0.2% Tween-20 and 10 μg/ml heparin (PTwH), the embryos were incubated with rabbit polyclonal anti-tyrosine hydroxylase antibody (1:300, Millipore, Dundee, UK AB152) in PTwH containing 5% DMSO and 3% donkey serum for 72 h at 37 °C. Following extensive washing in PTwH, the samples were incubated with donkey anti-rabbit 647 Alexa Fluor secondary antibody (1:300, Life Technologies, Paisley, UK, A-31573) in PTwH plus 3% donkey serum 72 h at 37 °C. After further washing in PTwH, samples were cleared by overnight incubation in tetrahydrofuran, followed by dichloromethane treatment for 15 min. The samples were placed in dibenzyl ether (DBE) until clear and imaged while submerged in DBE in 3D-printed slide chambers using a Zeiss LSM710 confocal microscope.

Quantification was performed using Fiji-Image J. Images were converted to greyscale and the Feature Extraction (FeatureJ Hessian) tool was employed using the smallest eigenvalue of Hessian tensor with the smoothing scale set to 0.5. To ensure consistent analysis across all conditions, multiple images from all mice were initially analysed to generate a uniform threshold value that was applied to every image analysed. A user-defined macro was used to provide a quantitative measurement of TH immunoreactivity within the midbrain dopaminergic projections. The data are expressed as a percentage of the mean of the wild type data. All imaging and quantification was performed blind with genotypes determined after TH quantification.

## 3 Results

### 3.1 APRIL enhances axon growth from midbrain dopaminergic neurons but does not affect survival

The dopaminergic neurons of the substantia nigra pars compacta (SNc) comprise the majority of dopaminergic neurons of the midbrain. They are the first dopaminergic neurons born, with a peak of neurogenesis at E11.5 in the mouse embryo. Axons initially embark upon a dorso-caudal trajectory before deflecting rostrally towards the striatum, reaching and beginning to ramify within this target field by E15.5 (Prestoz et al., 2012). To determine whether APRIL affects neuronal survival and/or axon extension we established dissociated cultures from ventral midbrain tissue at stages during the period axons are growing towards their striatal targets *in vivo*. To identify dopaminergic neurons in these cultures, we used immunocytochemical localisation of tyrosine hydroxylase (TH), the rate-limiting enzyme in dopamine synthesis. For experiments to assess the potential effects of APRIL on axon growth, it was necessary to prevent apoptosis under all experimental conditions in order to exclude the possibility that any differences in neuronal survival could confound the results. This was done in two ways, either the irreversible pan-caspase inhibitor Boc-D-FMK was added to the culture medium or the cultures were set up from mice that are homozygous for a null mutation in the *Bax* gene (Deckwerth et al., 1996). In addition to setting up cultures with and without APRIL, we also set up cultures supplemented with GDNF as a positive control because GDNF has been shown to enhance axon growth from cultured midbrain dopaminergic neurons and to enhance the survival of these neurons *in vitro*.

**Fig. 1:**
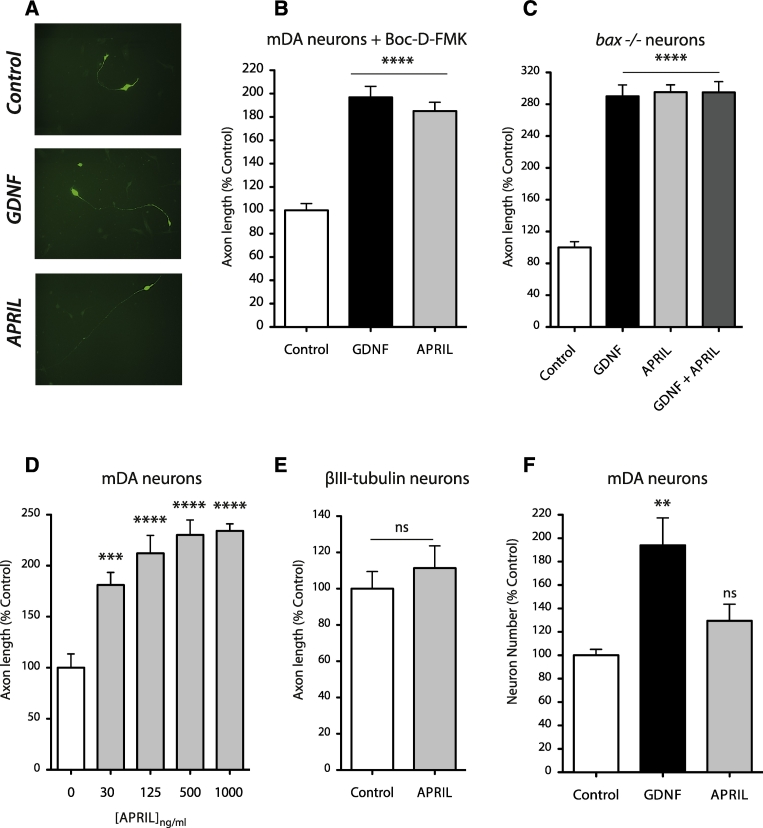
APRIL enhances axon growth from midbrain dopaminergic neurons but does not affect their survival. (A) Photomicrographs of representative E12 midbrain dopaminergic (mDA) neurons labelled with anti-TH after 48 h incubation under control conditions and in cultures supplemented with either 1 μg/ml APRIL or 20 ng/ml GDNF. (B) Axon lengths of E12 mDA neurons after 48 h incubation in cultures supplemented with 25 μM Boc-D-FMK. (C) Axon lengths of mDA neurons established from *Bax* −/− mice after 48 h in culture. (D) Axon lengths of mDA neurons cultured for 48 h in Boc-D-FMK-supplemented medium containing different concentrations of APRIL. (E) Axon lengths of midbrain neurons labelled with anti-βIII-tubulin after 48 h. (F) Survival of mDA neurons after 48 h under control conditions and with either GDNF or APRIL. All data are expressed as a percentage of the mean control value. Mean ± S.E.M. data of > 150 neurons per condition combined from 3 to 5 experiments of each type, **p < 0.01, ***p < 0.001, ****p < 0.0001, ns, not significant, ANOVA with *Bonferroni* correction, statistical comparison with controls.

After 48 h, < 1% of the neurons in E12 cultures were stained by anti-TH and these labelled neurons displayed the characteristic unipolar morphology of early midbrain dopaminergic neurons (Fig. 1A). In cultures treated with Boc-D-FMK and in cultures of BAX-deficient neurons, APRIL caused a highly significant two-fold increase in axon length of dopaminergic neurons compared with control cultures (Fig. 1A, B and C). This was very similar to the axon-growth promoting effect of GDNF on these dopaminergic neurons, and there was no further axon growth in cultures treated with both APRIL and GDNF in combination (Fig. 1C), suggesting that APRIL and GDNF act on the same subset of dopaminergic neurons. Dose response analysis showed that APRIL significantly increased dopaminergic neuron axon length at the lowest concentration of APRIL used (30 ng/ml), and reached saturation at a concentration of 500 ng/ml (Fig. 1D). In addition to enhancing axon growth from dopaminergic neurons, we investigated whether APRIL affects axon growth from other kinds of midbrain neurons. Using anti-βIII-tubulin to label all neurons, we found no significant difference in axon length between control and APRIL-supplemented cultures (Fig. 1E), suggesting that APRIL does not affect axon growth from the great majority of midbrain neurons.

In marked contrast to the similar axon growth-promoting effects of APRIL and GDNF on dopaminergic neurons, these factors had quite different effects on survival. Whereas GDNF significantly increased the survival of dopaminergic neurons compared with controls, there was no significant difference in the numbers of dopaminergic neurons surviving in cultures containing APRIL and control cultures (Fig. 1F). Taken together, these findings suggest that APRIL enhances axon elongation from developing midbrain dopaminergic neurons without affecting survival.

### 3.2 APRIL enhances neurite growth over a restricted period of embryonic development

**Fig. 2:**
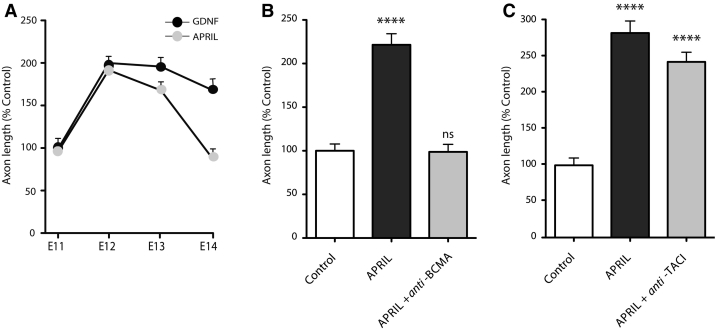
Developmental time-course of effect of APRIL axon growth and its mediation by BCMA. (A) Graph of axon lengths of mDA neurons after 48 h incubation in cultures supplemented with either 1 μg/ml APRIL or 20 ng/ml GDNF plotted as a percentage of axon length in control cultures at each age. (B) Bar chart of axon lengths of E12 mDA neurons after 48 h incubation in control cultures and cultures supplemented with either 1 μg/ml APRIL or 1 μg/ml APRIL plus 1 μg/ml function-blocking anti-BCMA antibody. (C) Bar chart of axon lengths of E12 mDA neurons after 48 h incubation with either 1 μg/ml APRIL or 1 μg/ml APRIL plus 1 μg/ml function-blocking anti-TACI antibody. All cultures received 25 μM Boc-D-FMK. Mean ± s.e.m. data of > 150 neurons per condition combined from 3 to 5 experiment of each kind, **p < 0.01, ***p < 0.001, ****p < 0.0001, ANOVA with *Bonferroni* correction, statistical comparison with controls. The increases in axon length were highly significant (p < 0.0001 in GDNF-treated cultures at E12 and greater and in APRIL-treated cultures at E12 and E13).

To ascertain whether APRIL enhances axon growth from midbrain dopaminergic neurons during a particular period of their development, we studied the effect of APRIL on axon growth in cultures established over a range of ages. As positive control, GDNF was included in these studies, as this factor enhances axon growth over an extended period of development. Neither APRIL nor GDNF promoted significant increases in axon length in E11 cultures. In E12 and E13 cultures, both APRIL and GDNF promoted significant increases in axon length compared with control cultures. However, by E14 whereas GDNF continued to enhance axon growth, APRIL had no effect on axon growth (Fig. 2A). These studies clearly show that APRIL enhances axon growth from midbrain dopaminergic neurons over a restricted period of development when these axons are growing towards their striatal targets.

### 3.3 APRIL enhances axonal growth from midbrain dopaminergic neurons *via* BCMA

APRIL exerts its effects in the immune system *via* two members of the TNF receptor superfamily, BCMA (TNFRSF13A) and TACI (TNFRSF13B) (Bossen and Schneider, 2006). To ascertain which receptor mediates the axon growth-promoting effect of APRIL, we investigated the influence of function-blocking antibodies to BCMA and TACI on APRIL-promoted axon growth. In these experiments, anti-BCMA completely inhibited the axon growth-enhancing effect of APRIL (Fig. 2B). Although anti-TACI caused a small reduction in APRIL-promoted axon growth (Fig. 2C), this reduction was not statistically significant. These results suggest that the axon growth-promoting effect of exogenous APRIL is mediated either exclusively or predominantly by BCMA.

### 3.4 APRIL and BCMA expression

**Fig. 3:**
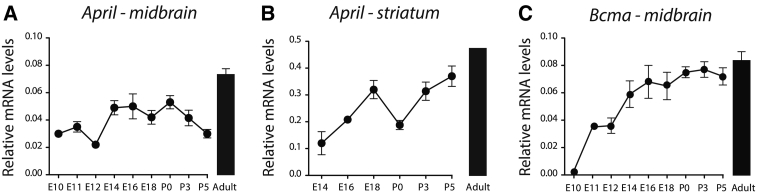
Developmental changes in the expression of *April* and *Bcma* mRNA in the midbrain and striatum. (A) *April* mRNA in the midbrain, (B) *April* mRNA in the striatum and (C) *Bcma* mRNA in the midbrain relative to the geometric mean of reference mRNAs. The mean S.E.M. of data from between three and six separate sets of tissues at each age are plotted.

To ascertain the developmental time-course of APRIL and BCMA expression, we used qPCR to measure the relative levels of transcripts encoding these proteins over a range of ages. *April* mRNA was detected in both the ventral midbrain and striatum. In the ventral midbrain, *April* mRNA was detected from the earliest age this region could be confidently dissected, E10, and its level relative to reference transcripts encoding housekeeping proteins remained similar throughout development (Fig. 3A). In the striatum, *April* mRNA was detected at the earliest age this could be confidently dissected, E14, and there was an overall three-fold increase to postnatal stages (Fig. 3B). In contrast to *April* mRNA, *Bcma* mRNA was undetectable in the ventral midbrain at E10. It was first detected at E11 and displayed a gradual increase throughout development (Fig. 3C). These results are consistent with the ability of ventral midbrain dopaminergic neurons to respond to APRIL in a BCMA-dependent manner and raise the possibility that these neurons obtain APRIL *in vivo* both locally and as a target-derived factor. Unfortunately, we were unable to study the cellular origin of APRIL synthesis *in vivo* because a highly specific antibody to APRIL for immunohistochemistry whose staining is eliminated in *April* −/− mice was unavailable.

### 3.5 Decreased density of midbrain dopaminergic neuron projection to the striatum in *April* −/− mice

**Fig. 4:**
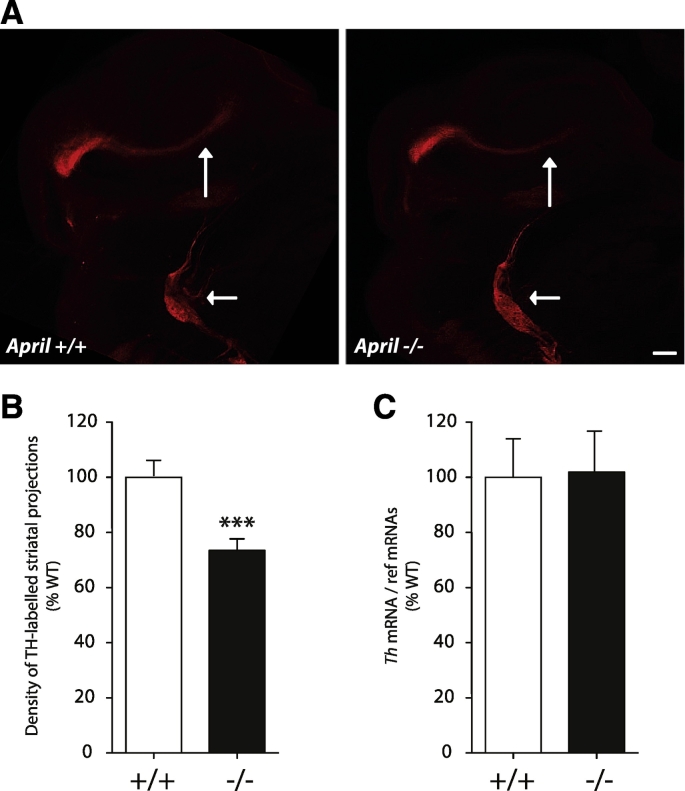
Decreased density of midbrain dopaminergic neuron projections to the striatum in *April* −/− mice. (A) Representative iDISCO images of the nigrostriatal projection and SCG stained with anti-TH antibodies in E14 *April* +/+ and *April* −/− mice. Large vertical arrows, nigrostriatal projection; Small horizontal arrows, SCG. Scale bar = 200 μm. (B) Quantification the density of TH-positive striatal projections in *April* +/+ (n = 20 embryos) and *April* −/− (n = 23 embryos) mice. (C) Levels of *Th* mRNA in the SCG of P5 *April* +/+ and *April* −/− mice. The mean S.E.M. of data from ten separate sets of tissues of each genotype.

To assess the physiological relevance of our *in vitro* findings, we compared the developing dopaminergic projection from the ventral midbrain to the striatum in *April* −/− embryos and *April* +/+ littermates. We studied TH-labelled dopaminergic striatal projections in iDISCO preparations at E14, by which stage axon outgrowth from the midbrain to the striatum is well established. These projections appeared more prominent in wild type embryos compared with *April* −/− embryos, whereas TH-positive superior cervical ganglia (SCG) and the fibre bundles that emerged from these ganglia were similar in both genotypes (Fig. 4A). To ascertain whether this impression was valid, we quantified the density of the striatal dopaminergic projection in *April* −/− embryos and in *April* +/+ littermates. Because the growth cones of individual dopaminergic axons could not be discerned with confidence, the length of developing striatal projection was not estimated. All quantification was done blind.

Quantification carried out on a very large number of embryos (*April* +/+ embryos, n = 20, *April* −/− embryos, n = 23) revealed a highly significant 29% reduction in the density of the midbrain dopaminergic projection in *April* −/− embryos compared with *April* +/+ embryos (p < 0.001) (Fig. 4B). To determine if this decrease in TH immunofluorescence was merely due to decreased neuronal expression of TH in the absence of APRIL, we measured the levels of *Th* mRNA in TH-expressing neurons in *April* +/+ and *April* −/− mice. Because the ventral midbrain lacks clearly defined anatomical borders, we did not use this structure for these studies because the size of dissected tissue is variable. Rather we measured *Th* mRNA levels in the superior cervical ganglion (SCG) which contains TH-expressing neurons and has clear anatomical boundaries that permit clean dissection from surrounding tissues. We found no significant difference in the levels of *Th* mRNA in the SCG of P5 *April* +/+ and *April* −/− mice (Fig. 4C), suggesting that APRIL does not regulate TH expression in developing neurons. Taken together, these findings suggest that APRIL plays a significant role in establishing the midbrain dopaminergic projection to the striatum during embryonic development.

## 4 Discussion

We have demonstrated that APRIL is a physiologically relevant trophic factor for developing nigrostriatal neurons. In culture, APRIL enhanced axon growth from midbrain dopaminergic neurons just as effectively as the established midbrain dopaminergic neuron trophic factor GDNF. Saturating concentrations of APRIL and GDNF in combination did not have an additive effect on axon growth, suggesting that they affect the same population of dopaminergic neurons. However, the action of APRIL and GDNF in culture differed in two respects. First, in marked contrast to GDNF, which enhanced axon growth and promoted the survival of midbrain dopaminergic neurons, APRIL enhanced axon growth without affecting neuron survival. Second, whereas APRIL enhanced axon growth over a restricted period of development, in cultures established at E12 and E13, GDNF enhanced axon growth in cultures set up at E12, E13 and E14. GDNF has also been reported to exert trophic effects on midbrain dopaminergic neurons at postnatal stages (Burke et al., 1998). These differences between GDNF and APRIL are similar to those observed in the developing peripheral nervous system (PNS) between classic neurotrophic factors and TNFSF members. Whereas neurotrophins and members of GDNF family promote both neuron survival and axon growth over extended periods of development (Davies, 2003), members of the TNFSF that affect the growth of PNS axons without affecting survival and do so during brief developmental windows (Gavalda et al., 2009, Kisiswa et al., 2013, McWilliams et al., 2015, O'Keeffe et al., 2008), with the exception of RANKL, which acts over an extended period of postnatal development (Gutierrez et al., 2013). While APRIL has been shown to enhance axon, but not dendrite growth, from cultured embryonic hippocampal pyramidal neurons (Osorio et al., 2014), it was not investigated whether the effects of APRIL on these neurons is restricted to a particular period of development and whether APRIL enhances the survival of hippocampal pyramidal neurons. Also, it is not known whether APRIL is physiologically relevant for the development of these neurons.

*In vitro* experiments using function-blocking antibodies against APRIL receptors suggest that BCMA, rather than TACI, mediates the effect of APRIL on axon growth from developing midbrain dopaminergic neurons. This is similar to the enhancement of axon growth by APRIL from cultured hippocampal pyramidal neurons, which is also blocked by anti-BCMA (Osorio et al., 2014). APRIL is unusual among the TNFSF in not being expressed at the cell surface as a membrane-anchored protein, but is processed in the Golgi apparatus by a furin-convertase enzyme to generate a biologically active, secreted protein (Lopez-Fraga et al., 2001). Because secreted APRIL can diffuse away from the cells that synthesize it, our demonstration that APRIL is expressed in both the midbrain and the striatum at the stage when midbrain dopaminergic neurons respond to the axon growth-promoting effects of APRIL *in vitro* suggests that these neurons may obtain APRIL *in vivo* both locally and from their targets.

In addition to GDNF, a large variety of neurotrophic factors have been reported to promote the survival of cultured midbrain dopaminergic neurons and promote neurite outgrowth from these neurons. These include the other members of the GDNF family, neurturin (Horger et al., 1998), artemin (Baloh et al., 1998) and persephin (Milbrandt et al., 1998), VEGF proteins (Piltonen et al., 2011), PDGF (Pietz et al., 1996), PACAP (Takei et al., 1998), members of TGFβ superfamily, including TGFβ 1, 2 and 3 (Krieglstein et al., 1995a), GDF5 (Krieglstein et al., 1995b) and GDF15 (Strelau et al., 2000), FGF20 (Ohmachi et al., 2003) and two more recently identified related factors CDNF and MANF (Lindholm et al., 2007, Petrova et al., 2003). Many of these factors have been reported to be protective for nigrostriatal neurons in animal models of Parkinson's Disease, including GDNF, neurturin, CDNF, MANF, PDGF, FGF20 and PACAP (Lindholm et al., 2016, Voutilainen et al., 2017). However, no obvious abnormal midbrain dopaminergic neuron phenotype has been reported in mice that lack any of these factors (Lindahl et al., 2017), and where an abnormal phenotype was initially reported in mice with a conditional deletion of *Gdnf* in the striatum (Pascual et al., 2008), comprehensive analysis of multiple mouse lines failed to find any abnormal dopaminergic phenotype (Kopra et al., 2015).

In contrast to the above studies, we show that APRIL-deficient mice display a defect in the developing nigrostriatal projection *in vivo*. The density of the dopaminergic nigrostratal projection, as visualized by TH staining in multiple iDISCO preparations, was significantly reduced in *April* −/− mice compared with *April* +/+ mice. This significant decrease was not due to a general decrease in TH expression in neurons that express this enzyme because there was no significant difference in *Th* mRNA levels in the SCG of *April* −/− and *April* +/+ mice. This decrease in the density of the dopaminergic nigrostriatal projection was quantified at E14, shortly after dopaminergic axons start growing to their targets in the striatum, which accords with the stage at which APRIL enhances the growth of axons from midbrain dopaminergic neurons *in vitro*. The shape and disposition of the nigrostriatal projection was similar in *April* −/− and *April* +/+ mice, suggesting that the guidance of these fibres is unaffected. In future work it will be interesting to ascertain whether this phenotypic change is sustained in older mice and how it might develop with age. It will also be important to ascertain whether there are secondary consequences for the maintenance of midbrain dopaminergic neurons and whether *April* −/− mice develop any motor defects with age. Finally, because degeneration of the dopaminergic nigrostriatal projection is a feature of Parkinson's disease, it will be informative to ascertain whether APRIL is efficacious in animal models of Parkinson's disease.

## Funding

This work was supported by grants 103852 and 086842 from the Wellcome Trust.

## Authors' contributions

TM instigated work and did the cell culture, LH did the *in vivo* analysis of midbrain dopaminergic projections, SW did the qPCR and AD supervised the work and wrote the paper.

## Conflicts of interest

The authors declare no conflicts of interest.
